# Hitting More Birds with a Stone: Impact of TGF-β on ILC Activity in Cancer

**DOI:** 10.3390/jcm9010143

**Published:** 2020-01-05

**Authors:** Cinzia Fionda, Helena Stabile, Cristina Cerboni, Alessandra Soriani, Angela Gismondi, Marco Cippitelli, Angela Santoni

**Affiliations:** 1 Department of Molecular Medicine, Sapienza University of Rome, Laboratory affiliated to Istituto Pasteur Italia—Fondazione Cenci Bolognetti, 00161 Rome, Italy; helena.stabile@uniroma1.it (H.S.); cristina.cerboni@uniroma1.it (C.C.); alessandra.soriani@uniroma1.it (A.S.); angela.gismondi@uniroma1.it (A.G.); marco.cippitelli@uniroma1.it (M.C.); 2 Neuromed I.R.C.C.S.-Istituto Neurologico Mediterraneo, 86077 Pozzilli (IS), Italy

**Keywords:** TGF-β, innate lymphoid cells, NK cells, TGF-β inhibitors, immunoevasion

## Abstract

Transforming growth factor (TGF)-β is a central immunosuppressive cytokine within tumor microenvironment inhibiting the expansion and function of major cellular components of adaptive and innate immune system. Among them, compelling evidence has demonstrated that TGF-β is a key regulator of natural killer (NK) cells, innate lymphoid cells (ILCs) with a critical role in immunosurveillance against different kinds of cancer cells. A TGF-β rich tumor microenvironment blocks NK cell activity at multiple levels. This immunosuppressive factor exerts direct regulatory effects on NK cells including inhibition of cytokine production, alteration of activating/inhibitory receptor expression, and promotion of the conversion into non cytotoxic group I ILC (ILC1). Concomitantly, TGF-β can render tumor cells less susceptible to NK cell-mediated recognition and lysis. Indeed, accumulating evidence suggest that changes in levels of NKG2D ligands, mainly MICA, as well as an increase of immune checkpoint inhibitors (e.g., PD-L1) and other inhibitory ligands on cancer cells significantly contribute to TGF-β-mediated suppression of NK cell activity. Here, we will take into consideration two major mechanisms underlying the negative regulation of ILC function by TGF-β in cancer. First, we will address how TGF-β impacts the balance of signals governing NK cell activity. Second, we will review recent advances on the role of this cytokine in driving ILC plasticity in cancer. Finally, we will discuss how the development of therapeutic approaches blocking TGF-β may reverse the suppression of host immune surveillance and improve anti-tumor NK cell response in the clinic.

## 1. Introduction

Transforming growth factor (TGF)-β is a potent immunosuppressive cytokine playing a key role in the regulation of cancer immunosurveillance. A large amount of this cytokine is produced and activated in the tumor microenvironment by many cell types, such as tumor, stromal, and immune cells (e.g., myeloid derived suppressor cells (MDSCs) and regulatory T cells (Treg)) [1]. 

Canonical TGF-β signaling is initiated by the engagement of monomeric type II receptors (TGFBRII), the recruitment of the serine and threonine kinase type I receptors (TGFBRI), and the assembly of a tetrameric receptor complex. In this complex, TGFBRI becomes active and phosphorylates SMAD2 and SMAD3 proteins, enabling them to interact with SMAD4 and to generate transcriptional complexes which bind to regulatory regions of many target genes. Moreover, non-canonical signaling pathways, such as MAP-kinases (p38, JNK, ERK) or PI-3 kinase, can contribute to TGF-β responses in different contexts [2]. Upon TGF-β stimulation, TGFBRI recruits and phosphorylates the adaptor protein ShcA to activate Ras at the plasma membrane and Erk1/2 [3]. Moreover, activated TGF-βR associate with and induce polyubiquitylation of the RING-domain E3 ligase TRAF6. Then, TRAF6 activates downstream kinases JNK, p38 MAPK, and IKK via TAK1 [4,5] but it can also induce ubiquitylation, membrane recruitment, and activation of Akt [6].

TGFBRI and TGFBRII are constitutively expressed on almost all cell types, but complex regulations at the level of ligand mobilization, receptor availability at the cell surface, and selective expression of SMAD protein interactors concur to define TGF-β responsiveness [7].

During tumor progression, TGF-β acts as a major inhibitor of adaptive and innate immune responses as well as a promoter of the function and the recruitment of immunosuppressive cells [8,9]. Among innate immune cells, TGF-β is considered to be an important regulator of innate lymphoid cell (ILCs) activity in cancer [10].

Innate lymphoid cells (ILCs) are a heterogeneous immune cell population which develops in the fetal liver and bone marrow from a common lymphoid progenitor, and typically lacks antigen-specific receptors and myeloid markers. Based on developmental and functional differences, ILCs are classified into five major groups: natural killer (NK) cells, innate lymphoid cell type1(ILC1s), type 2 (ILC2s), and type 3 (ILC3s), and lymphoid tissue-inducer cells (LTi) [11].

Natural killer cells circulate in the peripheral blood, but they are also found in several lymphoid and non-lymphoid organs, while other ILC subsets are mainly tissue resident cells.

The direct recognition of tumor cells by paired receptor–ligand represents a fundamental mechanism for NK cell activation and triggering of cytotoxicity and cytokine production. A wide repertoire of activating receptors mediates the binding to self-ligands highly expressed on tumor cells, triggering NK cell responses against these target cells. This group of receptors includes the natural cytotoxicity receptors (NCRs: NKp30, NKp44, NKp46) [12,13], the C-type lectin-like receptors (NKG2D (natural-killer group 2, member D) and CD94/NKG2C) [14], the activating forms of KIR, the adhesion molecule and co-activating receptor DNAM-1(DNAX accessory molecule-1) [15], and the co-receptors 2B4, CS1 (cell-surface glycoprotein CD2 subset 1), NTBA (NK-, T-, B-cell Antigen), and CD16, the receptor FcγRIIIA which binds the Fc fragment of IgG and mediates antibody dependent cellular cytotoxicity (ADCC) [16].

Differently, inhibitory receptors guarantee NK cell tolerance versus healthy cells via engagement of MHC-class I molecules or prevent NK cell-mediated killing of tumor cells which display elevated levels of other specific ligands. Inhibitory receptors include the killer cell immunoglobulin-like receptors (KIR), the heterodimer CD94/NKG2A, ILT2 (immunoglobulin-like transcript 2), PD-1 (programmed cell death protein 1), TIGIT, CD96, Tim3, and Lag-3 [17,18,19].

Non-NK cell ILCs are emerging as important players of anticancer immune responses [20,21,22], but their contribution has been established mainly in the context of infection diseases. Similarly, to NK cells, ILC1s produce IFN-γ and TNF-α providing protection against bacterial and viral infections [23,24]. Differently, ILC2s secrete IL-4, IL-5, IL-13, and amphiregulin and are involved in helminth control [25,26], while ILC3s have the capacity to produce IL-22 and IL-17 during fungal or bacterial infections [27].

Accumulating evidence suggest that functional plasticity is an important feature of ILCs, since appropriate environmental stimuli can modify their phenotypic and effector properties [28,29]. To this regard, a relevant immunosuppressive effect of TGF-β is due to its capability to induce the conversion of anticancer NK cells to pro-tumor ILC1s [10]. In addition, widespread evidence demonstrate that this cytokine dampens NK cell effector functions at multiple levels through inhibition of cytokine production and alteration of activating/inhibitory receptor expression leading to impaired cytotoxic activity [30,31,32,33]. Moreover, TGF-β deeply impacts on the phenotype of tumor cells to reduce their susceptibility to NK cell-mediated lysis as well as on tumor microenvironment to subvert anti-tumor immune response [9]. Therefore, it is clear that this cytokine exerts a profound effect on anti-tumor responses, already at the level of innate immunity.

Here, we summarize the current knowledge on the role of TGF-β in the regulation of ILC function in cancer. We focus on two major mechanisms whereby TGF-β strongly contributes to functional impairment of these immune effectors in tumor context: (1) regulation of NK cell activating/inhibitory signals; (2) guide of ILC plasticity. Moreover, as several drugs targeting TGF-β signaling are in development, we will discuss how these therapeutic approaches may reverse the suppression of host immune surveillance and improve anti-tumor ILC response in the clinic.

## 2. TGF-β Regulates NK Cell Activating and Inhibitory Signals in Cancer

**Figure 1:**
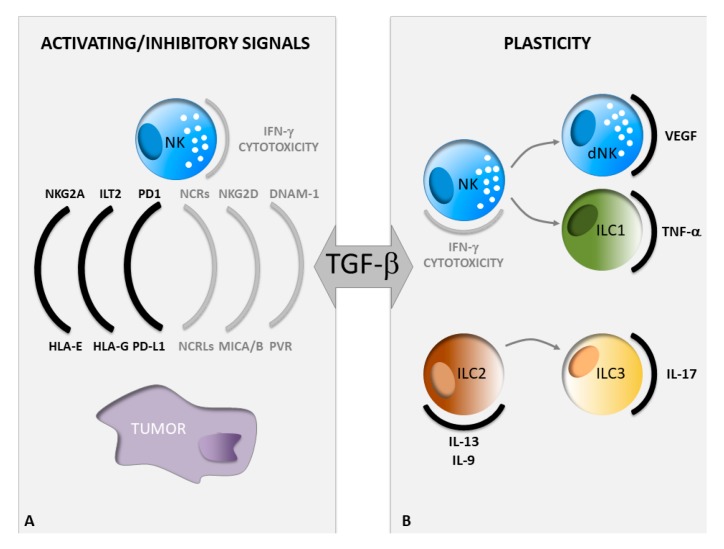
Impact of TGF-β on ILC functions in cancer. (**A**) Phenotypic changes of both NK and tumor cells in a TGF-β rich tumor microenvironment. The grey or black color indicates a decrease or an increase in the expression levels of the depicted molecules, respectively. (**B**) TGF-β-driven conversion of one ILC subset into another, ILC, innate lymphoid cells; NK, natural killer.

Impaired recognition of tumor cells by NK cells represents a critical mechanism of immune evasion. Indeed, the alteration of the NK cell receptor repertoire accounts for defective NK cell functions in cancer patients. Changes in the expression levels of NK cell activating or inhibitory ligands on tumor cells are also involved in rendering them less susceptible to NK cell attack [34]. In the tumor microenvironment, TGF-β gives a major contribution to these mechanisms. This cytokine exerts effects on both NK and tumor cells inducing profound phenotypic changes in these cells which compromise NK cell anti-tumor response. Below, we will report a detailed description of NK cell activating and inhibitory signals, mainly dependent on paired receptor/ligand interaction, which are significantly affected by TGF-β (Figure 1).

### 2.1. Regulation of NK Cell Activating Signals by TGF-β

The modulation of NK cell-activating receptor and ligand expression has a prominent role in the immunosuppressive function of TGF-β. The best characterized NK cell-activating receptor playing a prominent role in tumor immunosurveillance is NKG2D, whose ligands (MICA, MICB, ULBP1-6) are frequently induced on the surface of tumor cells in response to different types of cellular stress typical of a neoplastic transformation [35,36].

It has been widely reported that tumor-derived TGF-β could be the soluble factor responsible for the decreased NKG2D expression on NK cells and/or CD8+ T cells observed in several types of tumor patients [30,37,38,39,40,41,42]. In some of these studies, increased plasma levels of TGF-β inversely correlated with the expression of NKG2D, as well as of other activating receptors, such as DNAM-1, NKp30, and NKp46, on peripheral blood NK cells [30,37]. Moreover, ex vivo experiments showed that incubation of patient-derived NK or CD8+ T cells with plasma and/or cerebrospinal fluid obtained from patients, as well as with purified TGF-β, resulted in the selective down-regulation of the expression of these activating receptors [30,37]. Similarly, in a murine model of head and neck squamous cell carcinoma, tumors in the oral cavity as well as single tumor cell suspensions secreted high amounts of TGF-β. Incubation of IL-2 activated NK cells with tumor homogenate, cellular supernatant, or TGF-β alone significantly down-regulated NKG2D expression and inhibited killing of YAC-1 target cells [43]. However, suppressive functions of TGF-β could be exerted also when the cytokine is not released in its soluble form but associated to exosomes, nanometer-sized vesicles actively secreted by different cell types, including tumor cells. Cancer cell-derived exosomes expressing TGF-β can downregulate NKG2D expression on NK and CD8+ T cells, while other receptors, including CD4, CD8, CD56, CD16, CD69, and CD94 are not affected, indicating the selectivity of the effect [44,45,46,47].

Therefore, from all these studies, it appears that the immunosuppressive effects of TGF-β on NK and CD8+ T cells is partly dependent on NKG2D down-regulation. However, the presence of both TGF-β and soluble NKG2D ligands can have an even more significant impact on tumor progression and relapse. For example, in head-and-neck squamous cell carcinoma patients, the presence of high levels of both TGF-β and soluble MICA (sMICA) had a stronger influence on ex vivo IL-2 cultured NK cells as compared to patient plasma containing high sMICA and low TGF-β levels and were jointly responsible for inhibiting NKG2D expression and cytotoxicity [40,42]. Similar findings were obtained by the same group when they tested all the shed NKG2DLs (e.g., sMICB and sULBP1-3) [48]. The mechanisms of NKG2D ligand shedding have been largely investigated and are triggered by activation of metalloproteases (MMPs) [49], though it has been suggested that they are different from those activated by TGF-β [39,50]. This difference might be clinically relevant in relation to the possible future use of both TGF-β and MMPs inhibitors in therapeutic applications.

The molecular mechanisms responsible for the TGF-β-mediated down-regulation of activating receptors are still largely unknown, though some evidence suggests they can be at the transcriptional level. In fact, ex vivo treatment of NK cells with TGF-β alone or in combination with IL-2 decreased the NKG2D adaptor molecule DAP10 mRNA, by decreasing the association of RNA polymerase II with DAP10 promoter, while not significantly affecting NKG2D mRNA itself [51]. However, decreased NKG2D mRNA levels were observed in other experimental settings, where NKL cells were incubated with TGF-β, or with the supernatant of a glioma cell line [39]. Accordingly, TGF-β induced-miR-1245 was identified as a negative regulator of NKG2D by targeting the 3′UTR region of the *NKG2D* gene [52]. A significant decrease in transcript expression upon TGF-β treatment was observed not only for NKG2D, but also for NKp30, DNAM-1, granzyme B, and perforin, with a mechanism dependent on TGF-β-induced Smad2/3 signaling [33,53]. Moreover, TGF-β antagonizes the up-regulation of NK cell activating receptors induced by IL-15, as shown in an in vitro study analyzing NKG2D/DAP10, DNAM-1, and NKp30 expression. In this study, the IL-15-induced expression of multiple components of the NK cell cytotoxic machinery, including granzyme B, perforin, and cathepsin C was also affected [32]. However, the use of an IL-15 superagonist/IL-15 receptor alpha fusion complex (IL-15SA/IL-15RA) capable of activating the IL-15 receptor on NK and CD8+ T cells, was shown to be able to partially rescue the TGF-β-induced suppression of NK cell cytotoxicity, by interrupting Smad2/3-activity [53]. Restored expression of NKG2D, DNAM-1, and NKp30, as well as of granzyme A and perforin was observed also upon inhibition of Smad2 activation and TGF-β signaling by using the TGFRI kinase inhibitor Galunisertib [54] or an anti-TGF-β mAb (1D11) [55].

From a functional point of view, the most relevant consequence of TGF-β-mediated NKG2D downregulation is inhibition of cytotoxicity [30,39,43]. Interestingly, exogenous IL-15 can prevent both microvesicle-induced downregulation of NKG2D and impairment of NK cell cytotoxicity by interfering with SMAD protein activation. These observations provide a strong rationale for combined use of IL-15 and TGF-β blockade in immunotherapy [47]. Specific anti-TGF-β blocking antibodies or Galunisertib were widely used in these studies, being useful tools to demonstrate that NKG2D down-regulation is mainly mediated by this cytokine [30,32,37,39,46,47]. In one study, siRNA technology was also used as a possible therapeutic perspective to knockdown TGF-β1/2 expression [39]. In this study, the use of specific siRNA in glioma cells restored NKG2D expression on NK cell line NKL, upon co-culture with glioma-derived supernatants. Furthermore, TGF-β1/2 siRNA cells showed an increased expression of the NKG2D ligand MICA; higher levels of this ligand on cancer cells together with changes in NKG2D expression resulted in increased NK cell-mediated killing of silenced cells. In vivo, in an intracerebral glioma xenograft model (LNT-22 cells), TGF-β1/2 siRNA transfectants appeared to be non-tumorigenic and induced NK cell activation [39].

In summary, tumor-derived TGF-β severely affects the NKG2D-dependent anti-tumor immune response, by acting on both tumor and effector cells. In fact, it inhibits the expression of the ligands on one side, while on the other, it potentiates receptor down-regulation on various effector cells, particularly NK cells.

### 2.2. Regulation of NK Cell Inhibitory Signals by TGF-β

An efficient strategy to suppress NK cells is to shift the balance of signals governing their activity towards the inhibition. Indeed, increasing expression of inhibitory ligands on tumor cells and their paired receptors on NK cells is one of the mechanisms used by TGF-β to disrupt NK cell effector functions in cancer.

Among inhibitory ligands, several studies revealed that the non-classical HLA class I molecule HLA-G is a target of TGF-β. This molecule binds to the inhibitory receptors ILT-2, ILT-4, and killer Ig-like immunoglobulin receptor (KIR) 2DL4 and it is generally expressed by decidual trophoblasts and few other cell types; moreover, high levels of HLA-G characterize various types of malignant cells suggesting that expression of this ligand is one strategy used by tumor cells to escape immune surveillance [56,57].

In gastric cancer cells, TGF-β induces HLA-G expression through miR-152 inhibition, which results in the suppression of NK cell functions mediated by the interaction between HLA-G and the receptor ILT2 [58,59]. In agreement with this evidence, HLA-G induction is guided by TGF-β in ovarian cancer and in pancreatic adenocarcinoma cells where the cytokine increases also the surface levels of HLA-E, the ligand for the NK cell inhibitory receptor NKG2A [60,61]. These observations indicate that TGF-β can promote the delivery from tumor cells of various inhibitory signals towards NK cells. Indeed, the expression of LLT1 (Lectin-like transcript-1), the inhibitory ligand for C-type lectin NK cell receptor CD161, is regulated by TGF-β too. Stable knockdown of the cytokine as well as the exposure to the TGFBRI kinase inhibitor SD-208 leads to a down-regulation of cell surface LLT1 on glioma cells making them more susceptible to NK cell-mediated lysis; these observations indicate a key role for TGF-β in immunoevasive mechanisms in glioblastoma [62].

More importantly, TGF-β can impact on the inhibitory pathway mediated by interaction of PDL-1 ligand with the receptor PD-1, emerged as a major responsible for tumor immune escape [63,64]. In particular, it was recently reported that TGF-β elevated PD-L1 expression on cancer cells with negative effects on antitumor NK cell response. TGF-β-dependent expression of PD-L1 has a role in the mechanisms of drug-mediated resistance of tumor cells to NK cell cytotoxicity. Indeed, treatment with TGF-β signaling inhibitor SB525334 reverts the FASN (fatty acid synthase)-mediated resistance to NK cell cytotoxicity in cisplatin resistant lung cancer, probably through the modulation of PD-L1 levels on tumor cells. These findings suggest that the inhibition of FASN-TGF-β-PD-L1 axis may improve the efficacy of immunotherapy in treating cisplatin-resistant lung cancer [65].

In line with these observations, in breast cancer cells knockdown of Ly6E/K, the human homologue of the stem cell antigen-1 (sca-1), reduces both TGF-β induced-SMAD1/2 phosphorylation and surface expression of PD-L1 while increases binding of cancer cells to NK cells [66].

Interestingly, in non-small cell lung cancer, PD-L1 is also one of the molecules modulated during TGF-β-induced epithelial–mesenchymal transition (EMT), a key process in cancer progression in which epithelial cancer cells adopt a mesenchymal phenotype. Mesenchymalized tumors express augmented levels of PD-L1 and TGF-β may thus represent the molecular link between EMT and PD-L1 expression. In this context, different mechanisms of PD-L1 regulation may occur since TGF-β upregulates PD-L1 expression at the transcriptional level in a SMAD-2 dependent manner [67], whereas TGF-β-mediated mesenchymalization facilitates PD-L1 expression through epigenetic [68] and/or post-translational modifications [69]. Indeed, epigenetic modifiers, such as histone deacetylase or BET bromodomain inhibitors, are efficient to partially reverse TGF-β effects on EMT by decreasing PD-L1 expression too. Moreover, EMT may transcriptionally up-regulate N-glycosyltransferase STT3 through β-catenin, and STT3 further glycosylates and stabilizes PD-L1 protein [69]. In summary, these findings highlight that the regulation of PD-L1 expression on cancer cells may account for the immunosuppressive activity of TGF-β, providing a rationale, as discussed above, for double blockade of PD-1 and TGF-β signaling in anticancer therapy.

Modulation of inhibitory receptor expression on NK cells is an additional mechanism implicated in TGF-β-mediated tumor immunoevasion.

Importantly, TGF-β can control the expression of the inhibitory receptor CD96 on NK cells, breaking the balance between inhibitory, CD96 and TIGIT, and co-stimulatory CD226 receptors that share the common ligands CD155 and CD112. Indeed, the increased expression of CD96 by TGF-β on intra-tumoral NK cells of hepatocellular carcinoma patients leads to NK cell exhaustion with impaired cytokine production [70]. NK cell functional defects which accompany human breast cancer progression also correlate with an altered receptor repertoire as well as with high levels of TGF-β in the tumor microenvironment. In particular, NK cells from patients with different stages of breast cancer have a higher expression of the inhibitory receptors NKG2A and ILT2 as well as a lower expression of many activating receptors (NKG2D, DNAM-1, NKp30, and CD16), leading to a reduced cytotoxic activity. Blocking TGF-β produced by tumor cells can partially restore NK cell functionality [71].

Therefore, secretion of TGF-β in tumor microenvironment can sculpt the immune environment by modulating the inhibitory ligands on tumor cells as well as the inhibitory receptors on NK cells, and these changes could be predictive of a negative prognosis in cancer patients.

## 3. Guide of ILC Plasticity by TGF-β in Cancer

Despite the number of studies on the role of ILCs in cancer are progressively increasing, our knowledge on their impact on anti-tumor immune response remains very limited.

Indeed, an enrichment of selective ILC populations with altered activation status in tumor tissues and during cancer progression has been described in gastrointestinal, breast, lung, and other cancers, although their functional role remains less explored [72]. Moreover, a complex non-univocal behavior of these innate immune cells in the tumor context has been reported and the discovery of a great plasticity among ILCs, leading to the conversion of one population into another, further complicates our understanding of pro-tumor or anti-tumor properties of these lymphocytes [28,73]. Several soluble mediators govern the ILC plasticity within tissues in physiological conditions as well as during cancer development and progression. TGF-β has recently emerged as one of the key factors that play a role, not only in regulating ILC development and functions, but also in guiding the conversion among the different mature ILC populations [28,74,75] (Figure 1).

First evidence of NK cell functional plasticity in cancer highlighted the capability of TGF-β in the context of different advanced malignancies to induce a pro-angiogenic dNK cell phenotype of tumor infiltrating NK cells [76,77,78,79,80].

More recently, a novel mechanism of tumor evasion from immune control based on ILC plasticity and guided by TGF-β has been proposed by Gao et al., who described the TGF-β mediated conversion of NK cells into ILC1s in different tumor models [10]. In particular, this cytokine induces the generation from CD49a^-^CD49b^+^EOMES^+^ NK cells of CD49a^+^CD49b^+^EOMES^+^ intermediate type1 ILCs (intILC1) and CD49a^+^CD49b^-^EOMES^int^ ILC1s; interestingly, the frequency of these two last populations increases during cancer progression, while NK cell number declines. Although intILC1s share common features with both NK cells and ILC1s, they are endowed with higher expression of inhibitory receptors (CTLA-4, Lag3, CD96) and produce low amount of IFN-γ respect to TNF-α. Notably, tumor-infiltrating NK cells are the main producers of IFN-γ which is important in limiting tumor growth, while the high production of TNF-α by intILC1s and ILC1s can be responsible for the pro-tumorigenic and pro-angiogenic effects of these innate lymphocytes [81,82,83]. These findings are further supported by the evidence that SMAD4-deficient NK cells acquire an ILC1-like gene signature, losing their ability to control tumor growth and metastasis [84]. However, a TGF-β-independent role of SMAD4 in the control of NK cell functions was also demonstrated, since SMAD4 can directly regulate NK cell homeostasis, maturation, and cytotoxicity by targeting c-*kit*, *prdm1*, and *gzmb* genes [85]. We can suppose that the balance between the positive and negative role of SMAD4 towards NK cell anti-tumor functions may be due to the different TGF-β content of tumor microenvironments or to distinct stages of cancer progression.

The innate lymphocyte of type 2 resemble instead the innate counterpart of Th2 lymphocytes and it is well established that type 2 responses lead to tumor progression enhancing immunosuppression; consistently, the increase of ILC2 frequency observed in several cancers was associated to immunosuppression, tumor progression, and metastasis [72,86,87].

In a mouse model of breast cancer, tumor progression is associated with an increase of endogenous IL-33 and this cytokine promoted a significant rise in the amount of TGF-β-producing MDSCs within the mammary tumor. Notably, MDSC activity is tightly dependent on IL-13 and in this mouse model, IL-33 enhanced also the frequency of infiltrating IL-13-expressing ILC2s, suggesting a role of these lymphocytes in promoting the immunosuppressive functions of MDSCs [88]. Differently, an increased tumor growth was described in IL-9R deficient mouse; ILC2s together with Th9-cells are an important source of IL-9 in inflammatory conditions and TGF-β is able to induce IL-9 production by ILC2s, thus we cannot exclude that in the tumor microenvironment TGF-β-induced IL-9 release by ILC2s may elicit anti-tumor activity of these cells [89,90,91]. Recently, a crucial role for TGF-β in driving the trans-differentiation of ILC2s into ILC3s has been shown in inflammatory conditions [92]. In this context, TGF-β induced-IL-23R expression on a distinct subset of ILC2s expressing the c-kit receptor allowed these cells to respond to IL-23 and to produce the pro-inflammatory cytokine IL-17. Given the well-established association of inflammation with tumorigenesis, we can suggest a role for ILC2s-ILC3s conversion in cancer promotion [93].

Similarly to the type 1 and 2 ILCs, also for ILC3s pro- and anti-tumor functions have been reported [94,95]. However, no data are currently available on the impact of a TGF-β-rich tumor microenvironment on ILC3 functions and plasticity.

In conclusion, although we have made significant progress in understanding ILC biology, their impact on tumor development and progression is still controversial; a number of studies indicate TGF-β as a key regulator of these innate lymphocytes but additional studies are needed to clarify how this cytokine may impact on their functions and plasticity in cancer.

## 4. Targeting ILC Activity in Cancer via Inhibition of TGF-β Signaling

Despite the large amount of experimental in vitro and in vivo data concerning the wide spectrum of effects mediated by TGF-β in the tumor microenvironment, and in the regulation of the anticancer response mediated by various populations of effector lymphocytes, the development of therapeutic strategies able to inhibit TGF-β-mediated pathways did not proceed at the same speed as observed for other immuno-therapeutic approaches. Indeed, the initial evidence supporting a tumor-suppressor role for the TGF-β in several models, and the findings that first-generation TGFBR1 inhibitors triggered overt cardiac toxicity in preclinical studies [96], have greatly diminished the interests of pharmaceutical industry/research in this approach. Indeed, a major concern with strategies designed to modulate the activity of this pleiotropic cytokine is due to its widespread involvement in many normal physiological functions and this could generate important side-effects in vivo. Nevertheless, as already discussed above, it has become evident that genetic ablation of TGF-β signaling pathway components in selected immune cell types (e.g., CD4^+^ and CD8^+^ T cells, NK cells, or dendritic cells) can re-activate the anti-tumor response in different cancer pre-clinical models. In this context, several programs have been developed, aimed at inhibiting the activities mediated by this cytokine in cancer development and progression. Importantly, selected TGF-β transduction inhibitors revealed good safety profiles along with prolonged progression-free survival [96]. However, it will be important to investigate different biomarkers related to safety profiles during short dosing or longer treatments.

An increasing number of approaches, able to target various components of the TGF-β pathway, is currently being evaluated in clinical trials. In particular, they include: (1) direct inhibition of TGF-β production by antisense molecules; (2) blocking of TGF-β interaction with its receptor via monoclonal antibodies (e.g., mAbs against all three isoforms of TGF-β) or soluble decoy receptors; (3) small molecules/kinase inhibitors that interfere with the function of the SMAD signaling proteins (reviewed in [96]). Importantly, as TGF-β inhibitors are mainly targeting the tumor microenvironment, typically with modest direct effect on cancer cell proliferation, they should be used together with standard cytotoxic chemotherapy to efficiently kill these latter cells. Moreover, radiotherapy (and chemotherapy) can induce TGF-β activity, possibly supporting metastatic progression as side-effect activity [97,98]. In this context, combined TGF-β inhibition could improve tumor response to chemo-radiotherapy [98,99].

More specifically, some of the currently used clinical approaches are aimed at activating or enhancing the antitumor activity of NK cells. Moreover, promising results have been obtained by combined use of TGF-β inhibitors with other novel approaches for increasing NK cell anti-tumor immunity and expanding NK cells ex vivo.

Various TGF-β inhibitors are currently being tested in different cancers and investigated for their activity on NK cells, including Vactosertib (TEW-7197), an orally bio-available inhibitor of the TGFBRI, tested in phase I studies for advanced solid tumors (NCT02160106) or in association with the IMiD drug Pomalidomide for multiple myeloma (NCT03143985), where NK cells play a relevant role in the control of plasma malignant cells in the bone marrow. In this context, a different TGFRI kinase inhibitor, Galunisertib, has been investigated for AML and colon carcinoma and its administration has been associated with a significant up-regulation of the activating receptor NKG2D expression on NK cells and the release of TNF-α and IFN-γ [31].

Interesting results were also obtained using a recombinant chimeric protein termed FIST15, composed of the stimulatory cytokine IL15, the sushi domain of IL15Rα, and a TGF-β ligand trap [100]. In vitro FIST15 treatment enhanced the capacity of NK and CD8^+^ T cells to produce IFN-γ and TNF-α as well as to express cytolytic effector molecules, such as granzyme and perforin. More importantly, FIST15 predominately acted through NK cells to mediate control and clearance of B16-F0 melanoma tumor in vivo. This bifunctional biopharmaceutical, able to solve the problem of low clinical efficacy of cytokines caused by tumor immunosuppressive factors, has great potential as an immunotherapeutic agent.

More recently, it was demonstrated that TGF-β blockade can restore anti-tumor immune response also when combined with inhibitors of the immune checkpoint pathway PD-L1/PD-1. The fusion protein M7824, consisting of the extracellular domain of human TGFRII (TGF-β trap) linked to the C-terminus of human anti-PD-L1 heavy chain, significantly decreased tumor burden and increased survival in murine models of breast and colon carcinoma [101]. Antibody-mediated depletion of CD8^+^ T and NK cells was able to abrogate the M7824 activity supporting the anti-tumor role for these lymphocytes. Indeed, in tumor bearing mice treated with this bifunctional agent, tumor infiltrating CD8^+^ T and NK cells displayed a more active and cytotoxic phenotype.

In a different approach, genetic engineering was also used to generate stable SMAD3 silenced NK92 cells (NK-92-S3KD), the only FDA-approved human NK cell line for use in clinical trials. Knockdown of SMAD3 enhanced the cytotoxicity of NK92 cells against human hepatoma or melanoma cells as well as the production of IFN-γ via E4BP4, even in the presence of high amounts of TGF-β [102]. In line with these findings, the administration of NK-92-S3KD in NOD/SCID mice bearing human xenografts resulted in higher NK cell activation and cytokine production.

## 5. Conclusions

TGF-β has been demonstrated to be a key cytokine for tumor progression and evasion from immune surveillance. In particular, the importance of TGF-β as a mediator of impaired anti-tumor NK cell response is no longer debated. The role of non-NK cell ILCs in cancer are less clear and seem to vary greatly depending on the cytokine composition of the tumor microenvironment. In this scenario, the complex immunomodulatory activities of TGF-β play a major role for cancer progression given its profound effects on the activation, proliferation, and expression of inhibitory and activating receptors on these cells. Interestingly, a recent finding provides new insight into ILC1 plasticity in the tumor microenvironment, describing the TGF-β-driven conversion of NK cells into ILC1s, less efficient or unable to restrain tumor growth and metastasis in mice. This underscores a previously unknown mechanism by which tumors escape surveillance by the innate immune system. Further studies are needed to elucidate the contribution of specific ILC populations in cancer and to better understand whether the immunosuppressive activity of TGF-β may depend on its capability to modify phenotypic and functional properties of these effector lymphocytes. A better comprehension of these mechanisms will pave ways for anticancer therapeutic approaches targeting TGF-β signaling since their effects on non-NK cell ILCs remain unexplored.
